# Editorial: Aging-friendly environments and healthy aging

**DOI:** 10.3389/fmed.2023.1211632

**Published:** 2023-06-15

**Authors:** Yuling Jiang, Yifei Wu, Shaojie Li, Shihui Fu, Yuebin Lv, Hualiang Lin, Yao Yao

**Affiliations:** ^1^China Center for Health Development Studies, Peking University, Beijing, China; ^2^Department of Cardiology, Hainan Hospital of Chinese People's Liberation Army General Hospital, Sanya, China; ^3^Department of Geriatric Cardiology, People's Liberation Army General Hospital, Beijing, China; ^4^Chinese Center for Disease Control and Prevention, National Institute of Environmental Health, Beijing, China; ^5^Department of Epidemiology, School of Public Health, Sun Yat-sen University, Guangzhou, China

**Keywords:** aging-friendly environment, healthy aging, physical environment, social environment, health policy

## Introduction

Considering the rapid aging of the global population and the increasing burden of age-related diseases, it is imperative to identify modifiable risk factors for healthy aging and implement early interventions. Age-friendly environments play a crucial role in determining the quality of aging (1, 2). Extensive research has demonstrated that the aging process and the onset of diseases are influenced by complex interactions between individuals and their physical and social surroundings (3). These include factors such as neighborhood characteristics, exposure to indoor and outdoor air pollution, availability of social support, and access to the Internet. Notably, older adults are particularly susceptible to health issues resulting from adverse environmental conditions. Therefore, the objective of this Research Topic was to provide recommendations for future research and policy interventions that promote healthy aging and foster age-friendly environments. The Research Topic, titled “*Aging-friendly environments and healthy aging*,” encompasses 25 articles, primarily focusing on three key areas: the physical environment, the social environment, and the assessment and determinants of healthy aging.

## Physical environment

In this Research Topic, five papers investigated the associations between the physical environment and the health of older individuals, including extreme weather conditions, outdoor air pollution, and household/indoor air pollution. Vulnerable older populations face increased health risks due to climate change and ambient weather conditions. Lv et al. found that higher temperatures and lower relative humidity are linked to cataract formation in older adults, emphasizing the importance of addressing eye health issues related to drastic climate change in order to reduce the prevalence of cataracts and other eye diseases among older adults. Wang et al. demonstrate that both lower and higher ambient temperatures exacerbate pain perception in patients with disc herniation. These studies highlight the need to address climate change and enhance older adults' resilience to weather-related health risks to promote healthy aging.

Can environmental improvements effectively address cognitive impairment among older populations? A population-based, quasi-experimental study implemented China's Clean Air (CCA) initiative and suggested that it may mitigate the risk of air pollutant-associated cognitive aging among older adults (3). Zhang et al. indicated that an increase in green space coverage, an improvement in socioeconomic conditions, and increased participation in physical and social activities can benefit older adults' cognitive function. These studies provide scientific evidence that improving residential environments can potentially alleviate cognitive impairment in the future.

Furthermore, household air pollution (HAP) exposure resulting from the residential combustion of solid fuels remains a major health concern. Ma et al. examined the time trends of stroke mortality attributable to HAP in China and India. Their findings suggest that stroke death risks attributed to HAP have decreased in both countries, likely due to the reduction in the use of solid fuels, and this trend is also observed globally (4). However, there are still concerns regarding the uneven distribution of HAP-attributable strokes and the existing gap between the UN sustainable development goals on solid fuel use and the current situation.

Aside from HAP, indoor air temperature, lighting, and acoustics have an important impact on the health and quality of life of older people. Mu and Kang explored the relationship between the indoor environment of residential elderly care facilities in cold regions and the sensitivity of the old people to such facilities. The study findings indicated that participants were satisfied with the indoor environmental quality of the facilities, which was influenced by physical, environmental, and demographic factors. This study provides valuable insights for the design of other residential care facilities catering to older populations.

## Social environment

With the advent of the information age, the internet has become an important part of older adults' daily life and has also had a significant impact on their health. Li, Yue, Xiao found that Internet use may have a protective effect against mild cognitive impairment, potentially preventing cognitive decline in older individuals by influencing the volume of the globus pallidus. Similarly, Chen W.-c. et al. found that Internet use is associated with a positive impact on old people's self-rated health, physical health, and mental health. The COVID-19 pandemic has also stimulated interest in online health services among older people. Li, Shen, et al.'s study indicated that older adults expressed high satisfaction with the choice and management of medical resources through online platforms, influenced both by the pandemic's external factors and their own internal intentions. However, evidence has also shown that older individuals are more likely to face exclusion from the digital world, which is associated with poorer health outcomes (5). Therefore, in addition to highlighting the potential benefits of internet use for healthy aging, these studies suggest that governments and social institutions should improve elderly internet accessibility and enhance their use of technology to create a more digitally inclusive society.

Social support is another crucial factor in the health of older individuals. Li D. et al. examined the association between social support, physical and mental health, and the moderating effect of the community environment in Chinese older adults. Their study demonstrated that both formal and informal social support significantly influenced the physical and mental health of Chinese older adults. This research suggests the need for continued efforts to strengthen formal and informal social support provided to older adults and build a community-based care system that allows the community environment to moderate the relationship between social support and the health of older adults. Additionally, Cui et al. found that social support and health status also influenced the living arrangement preferences of older individuals, which is a crucial factor affecting their quality of life in late life.

Material and spiritual support from adult children is a vital component of social support necessary for older parents to cope with functional decline. According to Chen J. et al., contact with children (CCT) and satisfaction significantly predicted healthy aging. These findings highlight the importance of maintaining and increasing the frequency and satisfaction of parents' monthly contact with their children for healthy aging.

Government policies, such as vaccination campaigns during the COVID-19 pandemic, are well-known to protect the health of older individuals (6). Fan et al. provided evidence of the positive effects of the Urban and Rural Residents' Basic Medical Insurance (URRBMI) policy, which improved health outcomes for rural residents and reduced health disparities between rural and urban populations. Their study demonstrated that the implementation of the URRBMI policy contributed to promoting health equity. Another article by Tan et al. investigated Singapore's experience in developing regulations to govern assisted living facilities. Although still in the early stages of development in Singapore, assisted living is a viable care model that should be expanded to meet the increasing demand for care from the growing number of older populations.

## Assessment and determinants of healthy aging

According to the World Health Organization (WHO), healthy aging is defined as “the process of developing and maintaining the functional ability that enables wellbeing in older age” (7). However, there is currently no consensus on the measurement of healthy aging (8), and the determinants of healthy aging can vary depending on various factors (9).

Gao et al. have developed and validated a multidimensional population-based healthy aging scale (HAS), which included five dimensions: sensory capacity, cognitive capability, psychological capacity, locomotion capacity, and activities of daily living, which are validated to be reliable and valid measures for assessing healthy aging in older Chinese adults. This study provides a new tool to assess healthy aging in China. Another multidimensional disability indexes constructed by Han et al. include three aspects: individual, care resources, and social interactions. They found that among older adults living in communities, disability prevalence is high, and it has been associated with factors such as age, gender, education level, and chronic diseases. Thus, interventions that address these factors may be able to improve the functional ability and quality of life of older adults with disabilities in China.

Multiple determinants and potential pathways throughout the life course are associated with healthy aging. Two articles examine the prevalence of multimorbidity and frailty, which are critical risk factors for many age-related diseases. According to Chen S. et al., multimorbidity is prevalent among older Chinese adults, and the prevalence has more than doubled since 1998. It provided insight into the geographic distribution and temporal trends of multimorbidity in China and identified different patterns of multimorbidity. Li S. et al. studied the prevalence trajectory of frailty among older adults in China and analyzed the effects of age, period, and cohort. It was found that more recent cohorts exhibited a lower prevalence of frailty, indicating that social and environmental factors contribute to frailty development.

With the extension of global life expectancy and the increase in the older population, cognitive impairment and dementia have become serious problems worldwide. Finding modifiable intervention factors has become one of the key topics in the field of geriatric medicine. Li, Yue, Sun, et al. found that AST/ALT ratio may be a useful biomarker for predicting cognitive impairment in older adults based on three different clinical cohorts. Li and Yan found that older adults aged ≥60 years with napping < 30 min per day may be at lower risk of cognitive decline. This finding is useful for doctors to provide daytime napping recommendations for older people and help clinicians to identify the older people at risk of cognitive decline. Kim and Yeom investigated the detrimental impact of being underweight or overweight on cognitive function is heterogeneous by sex or cardiovascular risk. The findings from this study highlight useful assessment of BMI for groups of individuals who face a higher risk of cognitive decline from a change in BMI above the threshold. Moreover, Wu et al. found that declining cognitive trajectories were significantly associated with a higher risk of dementia and mortality. This study suggests that monitoring cognitive trajectories may be useful in identifying older adults at increased risk of adverse outcomes and intervening to improve their health.

Are cardiovascular risk factors and sleep complaints associated with insulin resistance (IR)? Podlipskyte et al. found that the major cardiovascular risk factors such as obesity, arterial blood pressure, and triglyceridemia, as well as sleep complaints, are more frequently observed in the IR group. Heimrich et al. explored interesting factors that determine why some older adults feel younger than their actual age. Several factors were identified as significant predictors of feeling younger in older adults, including more physical activity, a positive attitude toward aging, a higher standard of living, a better state of health, a higher level of satisfaction with life, and a better psychological wellbeing.

Falls prevention and bone mineral density (BMD) research in older people have also received attention. A prospective cohort study by Duan et al. aimed to explore the relationship between health-related physical fitness (HRPF) and falls. Based upon the findings of this study, the authors found that age, activity-specific balance confidence, and fitness abnormalities are all factors that contribute to the incidence of falls. Xiao et al. found that low-density lipoprotein cholesterol (LDL-C) levels were negatively correlated with lumbar bone mineral density (BMD) in young and middle-aged individuals, especially those individuals who were overweight and aged 30–49 years. The BMD of these individuals should be closely monitored and early intervention may be required.

## Perspectives

We summarized 25 articles across three domains: physical environment, social environment, and assessment and determinants of healthy aging. This Research Topic emphasizes the importance of constructing an aging-friendly environment and identifying as well as addressing modifiable risk factors for healthy aging. These papers provide valuable insight into the components of age-friendly environments and their impact on healthy aging outcomes. There is still a long way to go before we can create age-friendly environments and promote healthy aging for the entire population across the globe, given the substantial disparity in social development and health equity. The following recommendations may be helpful for future research:

- A comprehensive study of the environmental factors that contribute to healthy aging on both an individual level (e.g., genetics, lifestyle, and health behaviors) and at community level (e.g., socioeconomic status, social capital, and cultural norms). For the purpose of advancing age-friendly environments and promoting health in old age, more longitudinal observational and interventional studies are needed, which will provide higher levels of evidence. The cultural differences and health inequalities should be noted, which refer to differences in environmental factors between countries, such as the environmental factors present in countries with varying income levels.
- Establish evidence-based practices that can be adapted to different populations by developing reliable and valid measures evaluating the impact of aging-friendly environments on the health of older adults, as well as on wider societal outcomes, including healthcare costs and workforce participation.

Pay attention to the effect of cultural and social norms surrounding aging, such as stigma, ageism, and discrimination. It is crucial to address these biased attitudes and promote positive perceptions of aging in order to create an social environment that is age-friendly.

## Author contributions

YY, YJ, and SL: conception and design of the study and analysis and/or interpretation of data. YJ and YW: collating data and writing the original manuscript. YJ, YW, SL, YY, HL, YL, and SF: reviewing and editing the manuscript. All authors read and agreed to the published version of the manuscript.

## References

[1] Chen X Giles J Yao Y Yip W Meng Q Berkman L. The path to healthy ageing in China: a Peking University-Lancet Commission. Lancet. (2022) 400:1967–2006. 10.1016/S0140-6736(22)01546-X 36423650 PMC9801271

[2] National Programmes for Age-friendly Cities and Communities: A Guide. Geneva: World Health Organization; 2023. Licence: CC BY-NC-SA 3.0 IGO.

[3] Yao Y Lv X Qiu C Li J Wu X Zhang H. The effect of China's Clean Air Act on cognitive function in older adults: a population-based, quasi-experimental study. Lancet Healthy Longev. (2022) 3:e98–108. 10.1016/S2666-7568(22)00004-6 35224526 PMC8881012

[4] Lu H Tan Z Liu Z Wang L Wang Y Suo C. Spatiotemporal trends in stroke burden and mortality attributable to household air pollution from solid fuels in 204 countries and territories from 1990 to 2019. Sci Total Environ. (2021) 775:145839. 10.1016/j.scitotenv.2021.145839 33631580

[5] Lu X Yao Y Jin Y. Digital exclusion and functional dependence in older people: findings from five longitudinal cohort studies. EClinicalMedicine. (2022) 54:101708. 10.1016/j.eclinm.2022.101708 36353265 PMC9637559

[6] Wang G Yao Y Wang Y Gong J Meng Q Wang H. Determinants of COVID-19 vaccination status and hesitancy among older adults in China. Nat Med. (2023) 29:623–31. 10.1038/s41591-023-02241-7 36720270 PMC10285745

[7] Decade of Healthy Ageing: Baseline Report. Geneva: World Health Organization (2020).

[8] Michel JP Sadana R. “Healthy aging” concepts and measures. J Am Med Dir Assoc. (2017) 18:460–4. 10.1016/j.jamda.2017.03.008 28479271

[9] Abud T Kounidas G Martin KR Werth M Cooper K Myint PK. Determinants of healthy ageing: a systematic review of contemporary literature. Aging Clin Exp Res. (2022) 34:1215–23. 10.1007/s40520-021-02049-w 35132578 PMC8821855

